# Horizontal gene transfer and genome evolution in *Methanosarcina*

**DOI:** 10.1186/s12862-015-0393-2

**Published:** 2015-06-05

**Authors:** Sofya K. Garushyants, Marat D. Kazanov, Mikhail S. Gelfand

**Affiliations:** A.A. Kharkevich Institute for Information Transmission Problems, RAS, Bolshoi Karetny per. 19, build.1, Moscow, 127051 Russia; Faculty of Bioengineering and Bioinformatics, M.V. Lomonosov Moscow State University, Vorobievy Gory 1-73, Moscow, 119991 Russia

**Keywords:** Horizontal gene transfer, Archaea, Bacteria, Genome evolution, *Methanosarcina*

## Abstract

**Background:**

Genomes of *Methanosarcina* spp. are among the largest archaeal genomes. One suggested reason for that is massive horizontal gene transfer (HGT) from bacteria. Genes of bacterial origin may be involved in the central metabolism and solute transport, in particular sugar synthesis, sulfur metabolism, phosphate metabolism, DNA repair, transport of small molecules etc. Horizontally transferred (HT) genes are considered to play the key role in the ability of *Methanosarcina* spp. to inhabit diverse environments. At the moment, genomes of three *Methanosarcina* spp. have been sequenced, and while these genomes vary in length and number of protein-coding genes, they all have been shown to accumulate HT genes. However, previous estimates had been made when fewer archaeal genomes were known. Moreover, several *Methanosarcinaceae* genomes from other genera have been sequenced recently. Here, we revise the census of genes of bacterial origin in *Methanosarcinaceae*.

**Results:**

About 5 % of *Methanosarcina* genes have been shown to be horizontally transferred from various bacterial groups to the last common ancestor either of *Methanosarcinaceae*, or *Methanosarcina*, or later in the evolution. Simulation of the composition of the NCBI protein non-redundant database for different years demonstrates that the estimates of the HGT rate have decreased drastically since 2002, the year of publication of the first *Methanosarcina* genome.

The phylogenetic distribution of HT gene donors is non-uniform. Most HT genes were transferred from *Firmicutes* and *Proteobacteria*, while no HGT events from *Actinobacteria* to the common ancestor of *Methanosarcinaceae* were found. About 50 % of HT genes are involved in metabolism. Horizontal transfer of transcription factors is not common, while 46 % of horizontally transferred genes have demonstrated differential expression in a variety of conditions. HGT of complete operons is relatively infrequent and half of HT genes do not belong to operons.

**Conclusions:**

While genes of bacterial origin are still more frequent in *Methanosarcinaceae* than in other Archaea, most HGT events described earlier as *Methanosarcina*-specific seem to have occurred before the divergence of *Methanosarcinaceae*. Genes horizontally transferred from bacteria to archaea neither tend to be transferred with their regulators, nor in long operons.

**Electronic supplementary material:**

The online version of this article (doi:10.1186/s12862-015-0393-2) contains supplementary material, which is available to authorized users.

## Background

Horizontal gene transfer (HGT), also known as lateral gene transfer, plays a major role in the evolution of microbial genomes. It helps microorganisms to rapidly acquire new metabolic capabilities and adapt to environmental changes [1–3]. Most genes involved in HGT are associated with pathogenesis, symbiosis, metabolism, and antibiotic resistance [4].

HGT in bacteria is relatively well-studied and several estimates of its rate have been published [5–11], while horizontal transfer of genes from bacteria to archaea is less well characterized, but was shown to be important for origin of major archaeal clades [12].

HGT was shown to occur between both closely and distantly related organisms [13, 14]. According to the complexity hypothesis, horizontal transfer of genes encoding proteins with many protein-protein interactions is relatively infrequent as compared to HGT of genes encoding proteins with fewer interactions [15, 16]. Besides, genes tend to be transferred between genomes with similar codon usage [17].

HGT between bacteria and archaea was shown to be possible almost for all genes, except for a small fraction of genes toxic to the recipient organism [18].

*Methanosarcina* genomes are among the largest among archaea. It has been suggested that the large genome size in this genus is caused by massive HGT from bacteria [19]. Other members of the *Methanosarcinaceae* family are a psychrophile *Methanococcoides burtonii* [20], and halophiles *Methanohalobium evestigatum*, *Methanohalophilus mahii* [21], *Methanolobus psychrophilus*, *Methanosalsum zhilinae*, and *Methanomethylovorans hollandica*. All these species except the latter have much shorter genomes than *Methanosarcina* spp.

*Methanosarcinaceae*, like all *Methanosarcinales*, inhabit diverse environments and possess the largest set of metabolic pathways among Archaea. *Methanosarcinales* share such traits as acetoclastic methanogenesis, the presence of cytochromes, genes encoding the A, K, and N subunits of reduced coenzyme F420 (F420H2) dehydrogenase, bacterial-type phosphoglycerate mutase, bacterial adenylate kinase, nonhistone chromosomal protein MC1 involved in chromosome condensation, and the long variant of condensin subunit ScpB [22].

Horizontal transfer of a short operon from *Clostridia* [14, 23] was shown to dramatically change *Methanosarcinales* [14] or *Methanosarcinaceae* [23] metabolic capabilities, and allowed these organisms to use methyl compounds as substrates for methanogenesis. Strikingly, the first observations of possible HGT in *Methanosarcina mazei* showed that almost all functional types of genes could be horizontally transferred from bacteria to *Methanosarcina* spp. [19, 24, 25] and as much as 30 % of genes in *M. acetivorans* were predicted to be of bacterial origin [19]. About 50 % of genome of *M. burtonii* was shown to have atypical oligonucleotide composition and high transposon content, suggestive of HGT [20].

Analysis of specific patterns of gene gain in Archaea was performed using the arCOG database [26], and by phylogenetic tree reconstruction [27]. In both cases, some groups, and in particular *Haloarchaea*, *Methanomicrobia* and smaller taxonomic groups of methanogenic archaea were shown to acquire substantially more genes (more than a thousand for *Haloarchaea*) than others. While *Methanosarcinales* had been shown to acquire hundreds of metabolic genes from eubacteria by HGT [26, 27], the origin of such genes and their location in genome, as well as HGT events in the common ancestors of *Methanosarcinaceae* and *Methanosarcina* was not studied in detail. Here, we revisit the estimates of the HGT rate in *Methanosarcina* spp. and *Methanosarcinaceae*, and characterize possible donors of HT genes, their functions, operon structure, and gene expression.

## Results

### HGT identification

Groups of orthologous proteins (GOPs) were built for all *Methanosarcina* spp., as well as for all *Methanosarcinaceae* with similarity cut-offs 50 % and 40 %, respectively (Table 1).

**Table 1:** Statistics for initial orthologous groups in *Methanosarcinaceae*

		*Methanosarcinaceae* GOPs (3122)		*Methanosarcina* GOPs (2808)	
	# of proteins in the genome	# of proteins in initial GOPs	# of orphan proteins	# of proteins in GOPs	# of orphan proteins
*Methanococcoides burtonii* DSM 6242	2273	1811	462	−	−
*Methanohalobium evestigatum* Z-7303	2254	1699	555	−	−
*Methanohalophilus mahii* DSM 5219	1987	1713	274	−	−
*Methanosarcina acetivorans* C2A	4540	3325	1215	2852	1688
*Methanosarcina barkeri* str. Fusaro	3624	3062	562	2548	1076
*Methanosarcina mazei* Go1	3370	2888	482	2537	833

The number of GOPs in each category of groups is presented in brackets in the table header

All GOPs with more than half proteins annotated as transposases or transposon-associated proteins were removed from the data (30 *Methanosarcina* GOPs and 38 *Methanosarcinaceae* GOPs). The final database contained *Methanosarcina* GOPs that cover about 65 % of *Methanosarcina* proteins (2778 GOPs); 94 % (2624) of GOPs contained only one orthologous protein per organism; the rest contained paralogs (co-orthologs) for at least one species. From initial *Methanosarcinaceae* GOPs all GOPs that were already a part of *Methanosarcina* GOPs were removed, and only GOPs that have at least one protein from *Methanosarcina* spp., 55 % of the initial *Methanosarcinaceae* GOPs (1702; 9993 proteins from 6 species), were retained in the database. After that, 1375 *Methanosarcinaceae* GOPs included in the database did not contain paralogs.

**Fig. 1:**
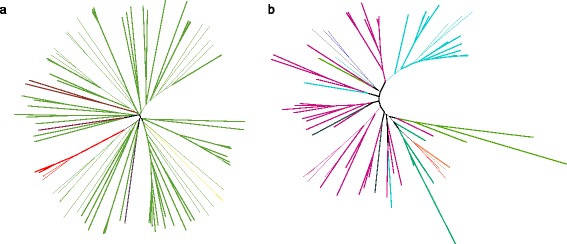
Phylogenetic trees for some GOPs. Phylogenetic trees constructed by maximum-likelihood algorithm for **a** acetate kinase AckA, involved in acetoclastic methanogenesis, and **b** uncharacterized alpha/beta hydrolase (PF12695) . *Methanosarcina* spp. are shown in red, other Methanosarcinaceae in orange, *Firmicutes* in light green, *Actinobacteria* in blue, *Synergistetes* in brown, *Thermotoga* in dark-purple, *Proteobacteria* in purple, *Dictyoglomi* in yellow, *Cyanobacteria* in emerald, and *Aquificae* in grey. Other archaea are shown in dark blue

All GOPs containing bacterial-type genes were selected using BLASTP (for details see Materials and Methods). For each selected GOP up to 100 top BLAST hits were aligned. Using this alignments, two sets of phylogenetic trees were constructed, neighbor-joining trees with bootstraps, and maximum likelihood trees, totaling 736 tree sets (Fig. 1a,b). If the *Methanosarcina* proteins were on the clade that contained *Methanosarcina* and bacterial proteins but no other archaeal proteins, the corresponding genes were considered to be laterally transferred from bacteria. Finally, we identified 349 *Methanosarcina* genes from 143 GOPs as likely laterally transferred from bacteria to the *Methanosarcina* last common ancestor. In addition, genes from 72 GOPs were transferred from bacteria to the last common ancestor of all *Methanosarcinaceae.* We further analyzed singletons (genes present in only one *Methanosarcina* spp.) and found that 14 genes were transferred recently in *M. acetivorans*; 33 genes were transferred in *M.barkeri*, and 10, in *M.mazei*. In *M. barkeri* we observed horizontal transfer of an operon comprised of four bacterial-type CRISPR-associated proteins (Additional file 1). Overall, 221 HT genes were found in *M. acetivorans*; 214 HT genes, in *M. barkeri*; 151, in *M. mazei* (Table 2 and Additional file 1). Hence, according to our estimates, about 5 % of genes in *Methanosarcina* were horizontally transferred from bacteria to either the last common ancestor of *Methanosarcinaceae* or *Methanosarcina*, or to one of the *Methanosarcina* species.

**Table 2:** Summary of predicted HT genes in *Methanosarina* spp

HGT in	# of GOPs	# of genes	Genes in MA	Genes in MB	Genes in MM
*Methanosarcinaceae*	72	178	66	53	59
*Methanosarcina*	143	351	141	128	82
HGT in one of the *Methanosarcina* species	-	57	14	33	10
All	215	586	221	214	151

MA – *M. acetivorans*, MB – *M. barkeri*, MM – *M. mazei*

The *M. mazei* genome is the smallest one in the genus, and only 82 genes in *M. mazei* remain that have been horizontally transferred to the last common ancestor of *Methanosarcina* spp. This is almost twice fewer than the number of genes transferred to the other two species (141 in *M. acetivorans*, 128 in *M. barkeri*)*.*

**Fig. 2:**
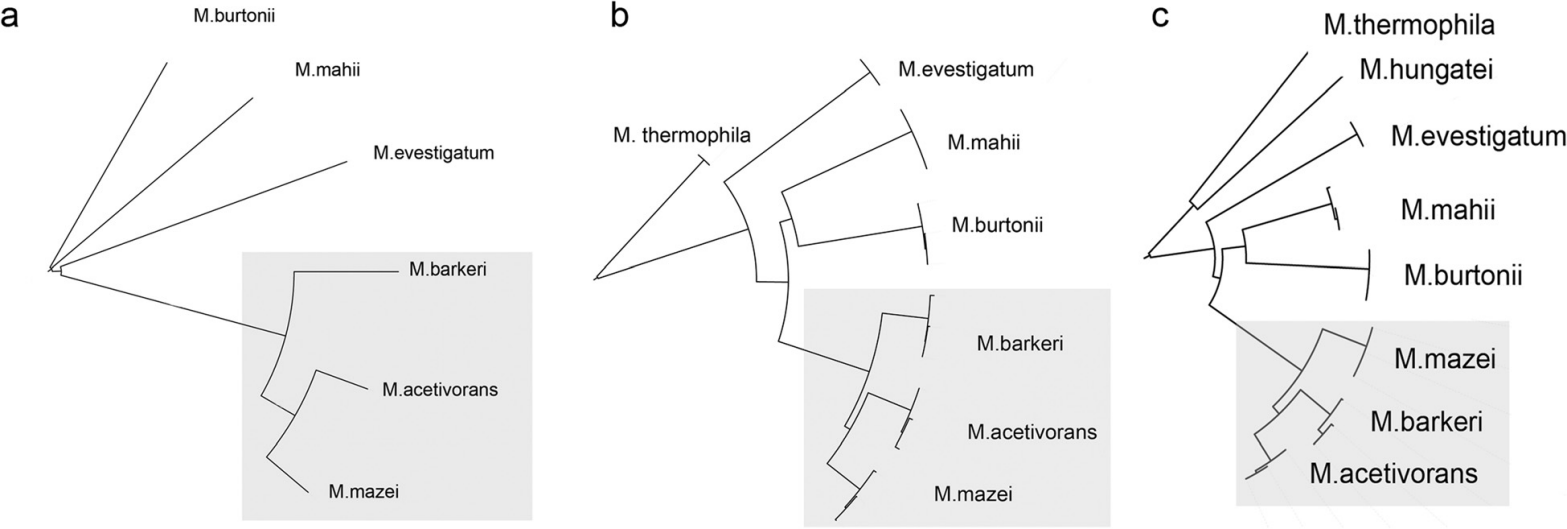
Phylogenetic trees for Methanosarcinaceae. Phylogenetic trees constructed by neighbor-joining algorithm are based on ribosomal proteins (**a**), 23S rRNA(**b**), and 16S rRNA(**c**)

The phylogeny of *Methanosarcinaceae* was reconstructed using 16S RNA [28, 29] and by conserved archaeal proteins [30]. These phylogenetic trees are not completely congruent. The phylogenetic trees based on proteins involved in translation and on 23S rRNA (see Methods, Fig. 2a,b) show that *M. mazei* and *M. acetivorans* are more closely related to each other than to *M. barkeri,* while the phylogenetic tree based on 16S rRNA clusters together *M. acetivorans* and *M. barkeri* to the exclusion of *M. mazei* (Fig. 2c). We found 12 GOPs containing HT genes only from *M. mazei* and *M. acetivorans*, and 67 GOPs containing HT genes only from *M. acetivorans* and *M. barkeri*, while no GOPs contained HT genes only from *M. mazei* and *M. barkeri*. If the 16S tree reflects the evolution correctly, then multiple HGT events had occurred in the common ancestor of *M.acetivorans* and *M. barkeri*, otherwise if the protein-based and 23S rRNA trees are correct, then *M. mazei* has lost a considerable fraction of genes horizontally transferred to the *Methanosarcina* last common ancestor.

### Time-scaled simulation of the non-redundant DB composition and the predicted HGT rate

**Fig. 3:**
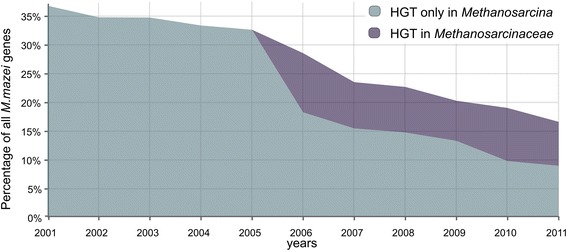
Relation between the estimated frequency of HGT and the year of DB release. To make the results consistent with the published data [17], HGT predictions were based on the results of BLAST search only, without subsequent tree construction, hence, the shown fractions for 2011 are higher than mentioned in the text. Blue area – HGT in *Methanosarcina* spp., purple – HGT in *Methanosarcinaceae*; see the text for definitions

The rate of the observed HGT to the last common ancestor of *Methanosarcina* or *Methanosarcinaceae* turned out to be drastically lower than that reported in previous studies [19, 25]. Two possible explanations for that may be, first, insufficient sensitivity of our procedure or, second, changes in the database composition. To select between these possibilities, HGT searches against a series of time-stamped databases were implemented. The number of bacterial genes in each database was fixed at the 2011 level, while the number of genes from archaea varied, so that, e.g., the 2001 database contained all archaeal genes sequenced strictly before 2002. In order to make the results consistent with the published data, all bacterial-like genes selected after the BLAST search were considered HT, without subsequent tree construction. The estimated fraction of candidate bacterial-origin genes in the last common ancestor of *Methanosarcina* spp. drastically decreases from 37 % (928 ORFs for *M. mazei*) as if in 2001 to 8 % in 2011 (222 ORFs for *M. mazei*). Additionally, 7.5 % of genes in 2011 (194 ORFs) seem to be horizontally transferred in the last common ancestor of *Methanosarcinaceae*. Figure 3 presents the data on *M. mazei* only, as in previous works the number of HGT events was calculated only for this species. Because in this experiment only genes in GOPs were analyzed, a smaller portion of HT genes in 2001 were obtained, 928 ORFs versus 1043 ORFs in previously published results [19].

The drastic change in the predicted rate of HGT to *Methanosarcina* is seen in the 2006 time-stamped database, when two *Methanosarcinaceae* genomes were sequenced, and events that could be earlier assigned as HGT to the last common ancestor of *Methanosarcina* turned out to be more ancient. Hence, the HGT effect on the *Methanosarcina* genomes after their divergence from other *Methanosarcinales* species probably has been overestimated in previous studies, and the HGT rate observed here seems to be more realistic. While the present analysis does not directly address the issue of the total number of genes of bacterial origin in the *Methanosarcina* genomes, this simulation demonstrates that it has likely been overestimated in early studies.

### The first control case: HGT in *Thermotogaceae*

*Thermotoga maritima* was also considered to be highly prone to HGT, as it had been claimed that 24 % of genes in these thermophilic bacterium had been transferred from archaea [31]. Later it was shown that the fraction of possible HGT genes in *Thermotoga* spp. is 8–11 %, but the BLAST e-value cutoff applied by the authors (10^−4^) was of low stringency [32]. We applied our BLAST-based pipeline to revisit these results. GOPs were built for proteins of all sequenced *Thermotogaceae*: *Thermotoga* spp., *Thermosipho* spp., *Petrotoga mobilis*, *Kosmotoga olearia*, and *Fervidobacterium nodosum*. All singletons and GOPs containing transposases were removed. Then for all GOPs that contained *T. maritima* proteins, the time-stamped pipeline was run, but in this case the numbers of both archaeal and bacterial genomes for each year-stamped database were changed. We found only 28 of 1761 analyzed GOPs (less than 1 % of all genes in genome) to be of possible archaeal origin, transferred to the common ancestor either of the genus or the family. This is smaller than the earlier estimates [31–34].

Then we repeated the procedure not for GOPs, but for all *T. maritima* proteins. When the 1999 database with the lowest cut-offs was considered, only 10 % (188 ORFs) of *T. maritima* genes were seen as possibly horizontally transferred, fewer than 451 in the original publication [31], and for the 2008 database this number is only 4.6 % (86 ORFs), again lower than the previously observed number of 204 ORFs [32].

Our possible explanation for this discrepancy could be that the 1999 study had considered individual genes, then constituting a large fraction of database entries, while we analyzed only complete genomes.

### The second control case: HGT in *Thermococcaceae*

To validate the approach against false positives we ran the HGT prediction procedure on sequenced *Pyrococcus* genomes. *Pyrococcus* spp. (family *Thermococcaceae*) are well-studied, and thought to have small genomes with a low rate of HGT. Indeed, the arCOGs analysis demonstrated massive gene gain in the last common ancestor of *Thermococcales*, but the origin of these genes was not discussed, although HGT from other archaeal clades was shown to be possible [26]. To identify possible events of HGT from bacteria, we applied the pipeline using the same settings as for the *Methanosarcina* spp. All *Pyrococcus* spp. genes were included in GOPs, no singletons were found, and 76 % of *Pyrococcus* GOPs contained exactly one orthologous protein per genome, while the rest contained paralogs in at least one species. One HGT event to the last common ancestor of *Thermococcaceae* involving a hypothetical protein was observed. The predicted low HGT rate in *Pyrococcus* spp. shows that the genomes of *Methanosarcinaceae* are indeed unusually dynamic.

### Taxonomic distribution of transferred genes

We further attempted to identify possible sources of bacterial genes horizontally transferred to the last common ancestor of *Methanosarcina* spp. or *Methanosarcinaceae. Firmicutes* and *Proteobacteria* were shown to be frequent donors of horizontally transferred genes: 104 GOPs contain genes transferred from *Firmicutes* with 12 of them further assigned to *Clostridia*, and 6, to *Bacilli*; 66 GOPs arose from *Proteobacteria*; 12, from *Planctomycetes*; 6, from the *Bacteroides/Chlorobi* group; 6, from *Actinobacteria*; 9, from *Cyanobacteria* etc. (Table 3). For 46 GOPs and 9 singletons, the origin could not be determined exactly. Though *Actinobacteria* is the third best-sequenced taxon, HGT from this group were rare and occurred only in the last common ancestor of *Methanosarcina*, moreover, all transferred genes were subsequently lost in *M. mazei*.

**Table 3:** Taxonomy distribution of horizontally transferred genes

Group	In *Methanosarcina*	In *Methanosarcinaceae*	All
*Firmicutes*	79	25	104
*Proteobacteria*	50	16	66
*unclassified bacteria*	27	28	55
*Planctomycetes; Planctomycetacia*	10	2	12
*Cyanobacteria*	7	2	9
*Bacteroidetes/Chlorobi group; Bacteroidetes*	3	3	6
*Actinobacteria*	6	0	6
*Synergistetes*	4	0	4
*Acidobacteria*	4	0	4
*Chlamydiae/Verrucomicrobia group*	3	0	3
*Thermotoga*	1	0	1
*Chloroflexi*	1	0	1
*Deferribacteres*	1	0	1
*Deinococcus-Thermus; Deinococci*	0	0	0

*Methanosarcina* in this case corresponds to either the last common ancestor of *Methanosarcina*, or even more recent HGT in one of the *Methanosarcina* species

**Fig. 4:**
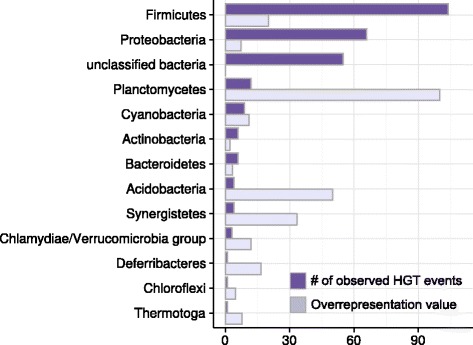
Taxonomic distribution of transferred genes. Distribution of HGT donors according to number of observed HGT events (purple) and overrepresentation values (lavender); see the text for definitions

As *Firmicutes* and *Proteobacteria* have the largest number of sequenced representatives, this can lead to overestimation of their effect. Indeed, it has been shown that the number of unique COGs increases with the addition of new organisms to the pan-genome [35, 36], so well-sequenced taxa have more unique genes in their pan-genomes. To offset that, the overrepresentation coefficient was calculated as the number of GOPs containing genes transferred from a given taxon divided by the number of sequenced genomes for this taxon (Fig. 4). This procedure implicitly assumed linear growth of the pan-genome size as new genomes are added to a taxon, which seems to be a correct approximation [35]. After applying this normalization we found that the most overrepresented taxa in the data are *Planctomycetes*, *Synergistetes*, and *Firmicutes*. Bacteria from these taxa as well as those from *Proteobacteria* and *Bacteroidetes*, co-occur in microbial communities with *Methanosarcina* spp. [37–39].

### Functional breakdown of the transferred genes

**Fig. 5:**
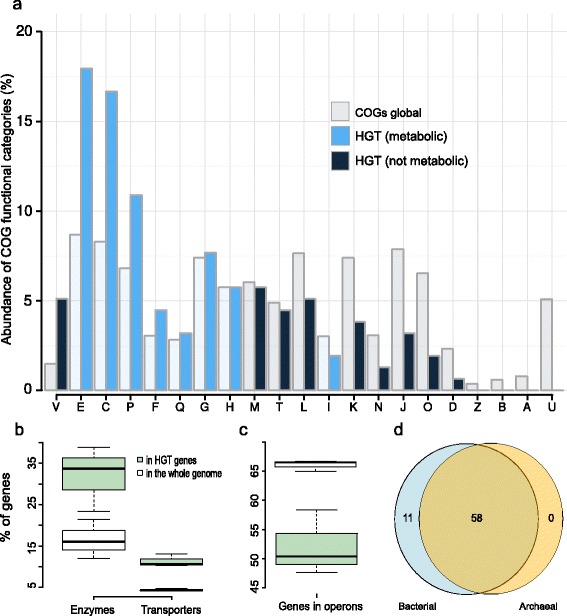
Functional distribution of transferred genes and their operon structure. **a** Representation of horizontally transferred genes in COG categories (see the text for definitions). **b** The fraction of genes encoding enzymes or transporters among the HT genes and the whole genomes. **c** The fraction of genes included in operons among the HT genes, and the whole genomes. **d** Venn diagram representing the distribution of COGs with transcriptional factors among Archaea and Bacteria

To characterize functional consequences of HGT, all identified HT genes were assigned to COG categories, 86 % (506 proteins) of HT genes had COG identifiers. Half of HT genes are associated with metabolism (Table 4). Assuming equal frequency of HGT for all COG categories, the expected transfer rates were based on mean fractions of the COG categories in all bacterial and archaeal genomes. Overrepresented categories include Defense mechanisms (V) and all types of metabolism except lipid metabolism and catabolism: Amino acid transport and metabolism (E), Energy production and conversion (C), Nucleotide transport and metabolism (F), Inorganic ion transport and metabolism (P), Carbohydrate transport and metabolism (G), Secondary metabolites biosynthesis, transport and catabolism (Q), Coenzyme transport and metabolism (H) (Fig. 5a).

**Table 4:** Functional distribution of HT genes

COG category		Presence in *Methanosarcina*						Presence in *Methanosarcinaceae*							
		No.	Enz.	Tr.	1	2	3	No	Enz	Tr	2	3	4	5	6
Information storage and processing:		18 (9,7 %)						7 (11,7 %)							
J	Translation, ribosomal structure and biogenesis	6	3	0	2	2	2	2	1	0	0	0	1	1	0
K	Transcription	7	1	0	5	1	1	3	0	0	0	0	0	2	1
L	Replication, recombination and repair	5	2	0	2	1	2	2	1	0	0	0	0	1	1
Cellular processes and signaling:		26 (14 %)						9 (15 %)							
V	Defense mechanisms	11	7	3	4	5	2	1	1	0	0	1	0	0	0
T	Signal transduction mechanisms	5	2	0	3	2	0	1	0	0	0	0	0	0	1
M	Cell wall/membrane/envelope biogenesis	8	1	0	4	1	3	5	2	2	0	1	2	2	0
N	Cell motility	1	1	0	0	1	0	0	0	0	0	0	0	0	0
O	Posttranslational modification, protein turnover, chaperones	1	0	0	0	1	0	2	0	0	0	0	1	1	0
Metabolism:		95 (51,3 %)						37 (54 %)							
C	Energy production and conversion	21	10	0	3	14	4	10	2	0	0	1	6	3	0
E	Amino acid transport and metabolism	25	10	8	1	13	11	9	5	1	0	0	2	1	6
F	Nucleotide transport and metabolism	7	5	0	1	4	2	3	2	0	0	0	0	0	3
G	Carbohydrate transport and metabolism	10	2	2	1	4	5	3	1	0	0	1	1	0	1
H	Coenzyme transport and metabolism	10	3	0	1	7	2	4	1	0	1	0	1	0	2
I	Lipid transport and metabolism	3	0	0	0	1	2	0	0	0	0	0	0	0	0
P	Inorganic ion transport and metabolism	16	2	9	2	9	5	6	0	5	0	0	0	1	5
Q	Secondary metabolites biosynthesis, transport and catabolism	3	0	0	0	2	1	2	1	0	0	0	1	0	1
Poorly characterized:		46 (24,8 %)						16 (23,5 %)							
R	General function prediction only	31	10	2	10	13	8	11	2	0	0	0	3	2	6
S	Function unknown	15	0	0	1	4	10	5	0	0	0	0	1	3	1

Tr. is the number of GOPs containing transporters, Enz. is the number of GOPs containing enzymes, numbers in the heading represent the number of genomes in GOP. All HT singletons were also included into this analysis

Then, HT proteins of *Methanosarcina* spp. were assigned to four major functional groups: housekeeping genes, enzymes, transporters, and transcriptional regulators. The first category comprises replication, translation and transcription machinery genes, as well as genes encoding cell wall proteins.

The number of transcriptional factors and house-keeping genes in our data was estimated using COGs, while the number of transporters and enzymes was estimated using PFAM and EFICAz databases, respectively.

Seven housekeeping GOPs with HT genes were found: t-RNA-dihydrouridine synthase; cysteinyl-tRNA synthase; two acetyltransferases or methyltransferases, whose function could not be characterized further; and three proteins involved in DNA repair. All other proteins involved in transcription, translation and replication were not found among the HT genes.

Among 226 transcriptional regulators observed in the *Methanosarcina* spp. only seven, belonging to two GOPs, were identified as HT ones. The fraction of transport proteins in *Methanosarcina* spp. comprises about 4.4 % of all genes (505 proteins), which is in good agreement with other estimates [40]. More than half of identified HT genes with an assigned functional class were either enzymes or transporters (187 and 67, respectively). The fraction of HT genes among transporters and enzymes was, respectively, 12-15 % and 9-11 % (Table 5). Hence, HGT of transporter genes is slightly more common than the transfer of enzyme genes, although this difference is not statistically significant (Table 5).

**Table 5:** Distribution of transporters and enzymes among HT genes and the genome in general

	Enzymes			Transporters			*χ*2-test
							p-value
	# of proteins in HT genes	# of proteins in the genome	% HT	# of proteins in HT genes	# of proteins in the genome	% HT	
*M. acetivorans*	86 (38.9 %)	971 (21.4 %)	8.9	29 (13.1 %)	194 (4.3 %)	14.9	0.0094
*M. barkeri*	50 (23.4 %)	435 (12 %)	11.5	22 (10.3 %)	172 (4.7 %)	12.8	0.6562
*M. mazei*	51 (33.8 %)	540 (16 %)	9.4	16 (10.6 %)	139 (4.1 %)	11.5	0.4663
All	187 (31.9 %)	1946 (16.9 %)	9.6	67 (11.4 %)	505 (4.4 %)	13.3	0.0163

Percent value in the brackets represents the % of enzymes or transporters in studied portion of genome (HT genes or all genes), while % HT column shows the percentage of occurrence of the proteins from this functional class among HT portion of genome. P-value is calculated for the null hypothesis that fractions of enzymes and transporters are equal among HT genes and for all genes

These results show that genes involved in metabolism, as well as in defense mechanisms (e.g. restriction-modification system, chloramphenycol O-acetyltransferase and multidrug transporters) are frequently transferred from *Bacteria* to *Archaea*. Lipid metabolism and catabolism is the only metabolic subsystem, where HT genes are rare. A natural explanation for that is the difference in the membrane composition of *Archaea* and *Bacteria* [41]. Overrepresentation of enzymes and transporters among HT proteins confirms that these types of genes are the most common subjects of HGT [9, 13, 35] (Fig. 5b). While HGT of both enzymes [14] and transporters [27] had been described in *Archaea*, the rates of their transfer were not compared directly.

### Operon structure

To characterize operons (for definition see Materials and Methods), all intergenic distances were calculated for each *Methanosarcina* genome. Initially six different intergenic spacers’ thresholds were analyzed (300, 250, 200, 150, 100 and 50 bp). A conservative value of 150 bp was selected as the maximum distance between genes in an operon, because with longer intergenic spacers, short HT operons start to merge with non-HT genes, while with a more stringent threshold on spacer length more than half of *Methanosarcina* genes are not included in operons.

About 67 % of *M. acetivorans* and *M. mazei* genes, and 65 % of *M. barkeri* genes comprise non-trivial operons. Of all operons with HT genes (Table 6), 10 operons are common for all *Methanosarcina* spp. For HT genes, the fraction of genes in operons is slightly lower, significant at *p* = 0.005 level (Table 6 and Fig. 5c). The average size of an operon with HT genes is 3.5 genes per operon, while the average operon length in the genome is slightly larger, comprising 4.1 gene per operon. 50 *M. acetivorans* operons, 32 in *M. barkeri*, and 33 in *M. mazei* were formed by both HT and archael genes. The mean length of a HT fragment is about 1300 nucleotides (see Materials and Methods), while the longest transferred segment consists of more than 7000 nucleotides, and contains 9 HT Na-ATPase genes. They form one operon, common for *M. acetivorans* and *M. barkeri*, and previously shown to be transferred as a single HGT event [42].

**Table 6:** Summary of the operon structure in *Methanosarcina* spp. among HT genes and for the genome in general

	Genome in general				HT genes				*χ*2-test
	Single genes	Genes in operons	# of operons	%	Single genes	Genes in HT operons	# of operons	%	p-value
*M. acetivorans*	1543	3063	768	66,5 %	92	129	80	58,4 %	0.0087
*M. barkeri*	1296	2402	570	65,0 %	112	102	61	47,7 %	5E-008
*M. mazei*	1145	2290	543	66,7 %	75	76	56	50,3 %	1E-005

HT operons are non-trivial operons with at least one HT gene. P-value is calculated for the null hypothesis that the fractions of operons and singletons are the same for HT genes and all genes

### HT genes expression

Available microarray and proteomics data were analyzed for three *Methanosarcina* species (see Methods). In four experiments on *M. mazei* [43–46], 82 of 151 predicted HT genes were shown to be differentially expressed under a variety of conditions (Additional file 1). Thirty of these genes were found to change the level of expression in the absence of histone; 66 genes were differentially expressed depending on the nitrogen source; 6 genes showed increased expression on methanol media; 10, increase on acetate media. Overall, in the *M. mazei* genome, 55 % of genes were shown to be differentially expressed, so the fraction of observed expressed HT genes is not lower than the genome average. In *M. acetivorans* proteomic data, 13 proteins (6.7 %) encoded by HT genes were found (for comparison, 9.1 % genes from the complete genome were identified) [47, 48]; in microarray data [49], 17 HT genes (12.6 %) showed differential expression on methanol or acetate as a food source, while the overall number of differentially expressed genes in this experiments reached 27.5 %. For *M. barkeri*, 7 HT genes (3.6 %) differentially expressed after air-exposure were found, whereas in the whole genome, 40 (1.6 %) such genes were observed [50]. Overall, 46 % of HT GOPs contain at least one member that was found to be differentially expressed. These results show that HT genes are active in *Methanosarcina* spp., and at least some genes have been shown to be differentially expressed, and hence are likely regulated.

## Discussion

HGT plays an important role in diversity and adaptation of microorganisms. In *Bacteria*, HGT initially was shown to be responsible for rapid spread of antibiotic resistance on plasmids [51]. Special gene transfer agents (GTAs) were discovered in the purple nonsulfur bacterium *Rhodobacter capsulatus* [52], and it was shown that in marine bacterial populations the transfer rate of antibiotic resistance genes included in GTAs was high [53]. Rapid gene acquisitions through HGT are thought to have driven adaptation to different ecological niches [1, 8, 54] and the origin of new bacterial and archaeal species [12, 14].

HGT rates for a variety of prokaryotic and eukaryotic [55] species were estimated. In particular, all members of the *Methanosarcina* genus were claimed to have dynamic genomes with a high HGT rate [19, 24, 25]. Later, HGT in *Methanosarcinales* was studied [26, 27], while for the family *Methanosarcinaceae* this phenomenon was not addressed directly, and the functional distribution of HT genes transferred to the last common ancestor of *Methanosarcina* spp. or *Methanosarcinaceae* was not investigated. We estimate that about 5 % of *Methanosarcina* genes are horizontally transferred from bacteria since the last common ancestor of *Methanosarcinaceae*. Among them, 3.5 % of genes were transferred to the last common ancestor of *Methanosarcina* spp. or even later in evolution, and 1.5 % of genes are the result of HGT to the last common ancestor of *Methanosarcinaceae*. Our estimate of the HGT rate in *Methanosarcina* generally agrees with the one provided by the arCOG analysis, where 321 HGT events were identified. However, in that study, the origin of the HGT was not identified [26].

In order to understand why the fraction of bacterial HT genes in *Methanosarcina* spp. has been overestimated in initial publications [19, 24], we performed searches through a series of time-scaled databases, and found that this effect was observed because of the database composition in 2001. Further, we re-evaluated the fraction of HT genes in *Thermotoga maritima*. Again, we could not reproduce the original result of 24 % HT genes [31], and observed a lower rate of 8-11 % [33]. Still, it looks like at least some HGT events from *Archaea* to *T. maritima* had occurred. We observed 28 such events, which is much lower than thought initially [31, 33, 34, 56]. In both cases, artifacts in previous estimates of the rate of gene flow between *Archaea* and *Bacteria* were likely due to incomplete and biased composition of available databases and application of simple BLAST-based procedures that were not sufficiently reliable given these biases. However, it is possible that more HGT events occurred earlier in the *Thermotoga* or methanogenic archaea evolution, and for *Methanosarcinales* a high rate of HGT from bacteria was shown [27].

Many HT genes common for *M. acetivorans* and *M. barkeri* are not present in *M. mazei*. On the phylogenetic tree constructed using ribosomal proteins, *M. acetivorans* and *M. mazei* form a cluster to the exclusion of *M. barkeri*. It means that *M. mazei*, whose genome is the smallest one among these three species, has lost many HT genes. While the last common ancestor of *Methanosarcina* spp. is thought to be a halophile as most *Methanosarcinaceae*, it could be speculated, that *M. mazei* gene loss could be associated with adaptation to low salt concentrations, one such example is the loss of Na + −ATPase and some metal transporters [42].

HGT played a major role in the development of acetoclastic methanogenesis. Examples of such transfer are genes coding proteins Pta and AckA, key proteins is acetoclastic methanogenesis, that were thought earlier, when no other *Methanosarcinaceae* genomes were sequenced except for *Methanosarcina* spp., were thought to be transferred to the last common of *Methanosarcinaceae* from *Clostridia* [14]. We found that only the clade with *Methanosarcina* proteins is situated within the *Clostridia* clade, while no orthologs of Pta and AckA have been found in other *Methanosarcinaceae* (Fig. 1a). This event had likely involved only the last common ancestor of *Methanosarcina* spp., while other *Methanosarcinales* use other methanogenesis enzymes.

In theory, HGT events can occur between any prokaryotic groups with similar codon usage [17, 18], but additional factors have to be considered, such as co-occurrence of organisms in the same ecological niches [57], or toxicity of HT gene products. The arCOG study did not reveal any ‘highways’ of HGT, that would preferentially connect particular groups of archaea and bacteria [58], but we have demonstrated that, at least for *Methanosarcina* spp., some trends may exist, and HGT from *Clostridia* and *Proteobacteria* to *Methanosarcina* spp. is the most frequent.

As *Clostridia* and *Proteobacteria* are the best sequenced groups of *Bacteria*, we also applied weighted measures to find the most frequent donors outside the best sequenced groups. Clearly, these calculations of overrepresentation are very approximate and have obvious limitations. Indeed, even in groups with hundreds of sequenced genomes, not all available ecological niches are sampled, and such groups often have numerous sequences for popular species. On the other hand, the complicated history of HGT in bacteria may further obscure the real donor. We presented both estimates with and without normalization for sequencing biases, and the truth probably lies somewhere in the middle. Still, while the most frequent donor could not be identified confidently, some robust trends could be observed, such as prevalence of *Firmicutes* as candidate donors.

We also considered the functional distribution of transferred genes. Both enzymes and transporters are the most frequent subjects of HGT (Fig. 5c). While transporters are more frequently transferred than enzymes, this observation does not reach statistical significance. However, this analysis is blind to archaea-specific transport proteins, that are still not well studied [40]. The addition of such transporters, as well as characterization of new classes of bacterial transporters, may influence the significance of this conclusion. Theoretically, the HGT of transporters is a convenient mechanism of adaptation to rapidly changing conditions, providing the cell with ions and nutrients from a new environment. ABC-transporters are known to be transferred between and within domains of life, and in a situation when such genes have been transferred from bacteria to *Methanosarcina* via a third, archaeal organism, whose genome is not available, false predictions could be made. Similarly, if a gene has been subject to HGT from *Methanosarcina* to another archaeal species, our study design would not identify it as transferred from bacteria to *Methanosarcina*. However, here we concentrated on recent HGT, where such misidentifications are less likely.

While the membrane of *Bacteria* and *Archaea* consists of different types of lipids, these results show that bacterial-type transporters could work in both cell types. The only class of transporters underrepresented among HT genes is lipid transporters, similarly to lipid metabolism enzymes, likely reflecting differences in the membrane structures.

The transfer of enzymes is rarer, mainly because a new enzyme has to be incorporated in a pre-existing metabolic pathway, and, further, subunits of protein complexes and proteins with multiple interactions seem to undergo fewer HGT events [15, 16].

HGT of bacterial genes has been studied for the last common ancestor of *Methanosarcinales* (three *Methanosarcina* genomes, *M. burtonii* and *Methanosaeta thermophila* RT) [7], and while the distribution of functional classes is similar with the one that we observe for HGT in the common ancestors of *Methanosarcinaceae* and *Methanosarcina*, there are some differences. Firstly, for *Methanosarcinales* more transfers of genes involved in replication, recombination and reparation (category L) have been observed; secondly, there are fewer transporters among transferred genes. The latter may be caused by difficulties in establishing orthology in large gene families, common for transporters, at large evolutionary distances. Also, for relatively recent events we observed that half of the transferred genes are involved in metabolism, while in the *Methanosarcinales* study [27] only 26 % of gains are associated with metabolism. Hence, the presented results show that relatively recent HGT events tend to impact the metabolic potential, but not the basic cell functions, such as replication or recombination.

More than a half of HGT genes found in *M. mazei* were shown to be functional and differentially expressed, and the important question is how all these genes are regulated. The total number of known transcription factors in *Methanosarcinales* corresponds to earlier predictions for prokaryotic organisms [59]. This observation may be biased by the fact that the number of transcription factors was estimated using the COG classification, that contains no clusters comprised exclusively by archaeal TFs, while many TF clusters are bacteria-specific (Fig. 5d). Only in two HGT events TFs were actually transferred, but about 46 % of GOPs were shown to be expressed, and if the regulators tend to be transferred together with the genes they regulate, we would expect to see more HGT events involving TFs. This leads to a conclusion that HT genes from bacteria do not tend to be transferred with their regulators, but are mainly regulated by the factors already existing in the acceptor organism.

As we have considered only recent HGT events, we do not expect massive genome rearrangements since HGT, and that has allowed us to analyze the operon structure of HT genes. Among HT genes, we have observed a lower fraction of operons, and the characteristic length of the transferred fragments is about 1200 bp (the average length of one protein-coding gene). Moreover, there are also mosaic operons comprised of both HT and archaeal genes. It is possible that the length of a HT fragment is limited by the mechanism of gene transfer from bacteria to archaea.

## Conclusions

Previously, the role of HGT in *Methanosarcina* spp. was overestimated due to biased data. A more robust estimate of the fraction of HT genes either in the last common ancestor of *Methanosarcina* spp. or of *Methanosarcinaceae* is ~ 5 %. We studied the operon structure of HT genes and showed that the HT genes do not tend to be transferred as whole operons. Most frequent HGT donors are *Firmicutes* and *Proteobacteria*. While the regulation of HT genes is not well understood, about half of identified HT genes in *M. mazei* are differentially expressed.

## Methods

### Genomes

Genome sequences were downloaded from Genbank (www.ncbi.nlm.nih.gov).

Four groups of species were used: (1) *Methanosarcina* spp. (*M. acetivorans* C2A (NC_003552; NC_002097) [24], *M. barkeri* str. Fusaro (NC_007349) [25], *M. mazei* Go1 (NC_007355; NC_003901) [19]); (2) all Methanosarcinaceae available as of March, 2012 (*Methanosarcina spp.*, *Methanococcoides burtonii* DSM 6242 (NC_007955) [20], *Methanohalobium evestigatum* Z-7303 (NC_014253), *Methanohalophilus mahii* DSM 5219 (NC_014002) [21]); (3*) Pyrococcus* spp. (*P. horikoshii* OT3 (NC_000961) [60], *P. abyssi* GE5 (NC_000868; NC_001773) [61], *P. furiosus* DSM 3638 (NC_003413) [62]); and (4) all available Thermotogaceae (*Thermotoga thermarum* DSM 5069 (NC_015707), *Thermotoga sp.* RQ2 (NC_010483.1) [63], *Thermotoga petrophila* RKU-1 (NC_009486.1) [33], *Thermotoga neapolitana* DSM 4359 (NC_011978.1), *Thermotoga naphthophila* RKU-10 (NC_013642), *Thermotoga maritima* MSB8 (NC_000853) [31], *Thermotoga lettingae* TMO (NC_009828)*, Thermosipho melanesiensis* BI429 (NC_009616), *Thermosipho africanus* TCF52B (NC_011653), *Petrotoga mobilis* SJ95 (NC_010003), *Kosmotoga olearia* TBF 19.5.1 (NC_012785) [64], *Fervidobacterium nodosum* Rt17-B1 (NC_009718)).

### Grouping of orthologs

Initially, groups of orthologous proteins (GOPs) were constructed for every pair of species in a group. All pairwise comparisons were done using BLASTP [65], and bidirectional best hits (BBHs) were identified. Hits were ignored if the identity level was less than 50 % (40 % for *Methanosarcinaceae*) or if the aligned region was less than 2/3 of the length of the shorter protein. Then, if two paralogous genes from one genome were more similar to each other than to a BBH partner from another genome, both were added to the orthology group. Then, maximal connected components were constructed. The groups were formed using ad-hoc software written using Oracle RDBMS Express Edition (PL/SQL codes are available in Additional file 2).

### Identification of HGT

To identify HGT events, several perl scripts were developed. Each member of a GOP was used as a query in a BLAST search against the non-redundant protein sequences database (release 2011-07-16) with default parameters. Organisms were classified according to the NCBI taxonomy [66].

If all proteins of an analyzed GOP had three top hits only in the *Bacteria* superkingdom or only three among twenty top hits were archaeal, while all others were bacterial (with identity cut-off at least 30 %, length of HSP not less than 50, and coverage of a *Methanosarcinaceae* protein by a bacterial hit not less than 75 %), the GOP was retained for further analysis.

At the next step, top 100 protein hits for each member of the GOP were selected. The selected proteins for all GOP members were aligned by MUSCLE (version 3.6) [67] with default settings. The alignment quality was controlled by GUIDANCE [68] and manually. If more than half of columns in an alignment had score less than default GUIDANCE cutoff (0.93), such GOPs were excluded from the analysis. For each alignment, two types of trees were constructed, a neighbor-joining (NJ) tree with 100 bootstrap replicas using the ClustalW software with default parameters (BLOSUM distance matrix) [69], and a maximum-likelihood (ML) tree using PhyML v3.0 with default parameters [70]. Trees were visualized using the iTOL server [71].

NJ and ML trees were analyzed independently. No special rooting procedures were applied, but if non-*Methanosarcinaceae* archaea formed a monophyletic clade on the tree, the root was placed manually between these archaea and bacteria. If no such monophyletic clade was present or only *Methanosarcinaceae* archaea were present, the root was placed in the point of divergence of large bacterial taxa. If for both NJ and ML trees all studied *Methanosarcinaceae* genes formed a stable subclade (for NJ tree with bootstrap > 70) within a bacterial clade, without other archaeal proteins, such GOPs were considered as candidate HGT. For each HGT event, organisms closest to *Methanosarcinaceae* on the tree were analyzed to identify the source and timing of the HGT event. It should be noted, however, that both individual decisions in each particular case and overall conclusions are robust as regards the exact position of the root, unless it is positioned on a branch between the *Methanosarcinaceae* clade and the rest of the tree.

A HGT event was considered to be a transfer to the last common ancestor of *Methanosarcina* spp., if all members of the *Methanosarcina* GOP, consisting of at least two *Methanosarcina* spp. proteins from different species, formed a stable clade only with bacterial proteins, and no other archaeal proteins were present on that clade, but they could be present elsewhere in the tree. HGT to *Methanosarcinaceae* was detected if all members of the *Methanosarcinaceae* GOPs (at least two proteins of *Methanosarcina* species, and least one protein from other *Methanosarcinaceae* species) are grouped with bacteria and not with other archaea. The source of HGT was identified as the lowest common taxonomic rank for all bacteria that form a stable clade with GOP members.

To analyze orphan genes not included in any GOPs and find recent HGT events in the *Methanosarcina* species the same procedure was applied.

The BLAST-based pipeline include only BLAST search, as described above, without further tree construction.

### Phylogenetic trees of *Methanosarcinaceae*

Concatenated alignment of ribosomal proteins S2, S5, L2, L3, L4, L6, two copies of S4, and protein EF-TuA as in [72] was generated using the MUSCLE software. The rRNA alignments were downloaded from the SILVA database [73]. These alignments were used to produce neighbor-joining trees with 100 bootstrap replicas by ClustalW software, as described above.

### Assigning proteins to COG

Each predicted HGT protein was assigned to an orthologous group in the COG IMG database [74]. For each studied organism, the overrepresentation (O) for a COG category was measured as:

$$ O=\frac{CO{G}_{obs}-CO{G}_{\exp }}{CO{G}_{\exp }}, $$

where *COG*_*exp*_ is the normalized number of COGs in the COG category among all archael or bacterial genes, and *COG*_*obs*_ is the number of observed COGs in this category among the HT genes.

### Assigning proteins to functional classes

The fraction of transcription regulators was estimated separately for each *Methanosarcina* species by calculating the number of proteins in the COG category “Transcription regulation”.

The fraction of transport proteins in *Methanosarcina* proteomes was estimated using PFAM [75, 76]. The total number of proteins assigned to PFAM families was calculated for each complete *Methanosarcina* proteome and also for those proteins that were clustered into GOPs. All *Methanosarcina* proteins were extracted from PFAM families with keyword ‘transport’ in the family name, and the fractions of transport proteins were calculated for proteins clustered into all GOPs, and for proteins clustered in HT GOPs. The fraction of enzymes in the complete proteome, in GOPs, and in HT GOPs was estimated by the EFICAz EC classification [77]. All *Methanosarcina* entries with EC annotation were extracted from the database and then enzyme functions were assigned to the HT genes and all proteins in the *Methanosarcina* GOPs using NCBI gi identifiers.

### Operon prediction (selection of parameters, comparison with microarray data)

All gene coordinates and directions were extracted from Genbank genome annotations. Intergenic distances were calculated using perl scripts. Operons were defined as sets of adjacent codirectional genes with short intergenic spacers, with the thresholds discussed in the Results section. The length of HT fragment was estimated by a sum of lengths of sequential HT genes and intergenic spacers between them. For trivial operons, the length of HT fragment equals to gene length. Singleton HT genes were excluded from this analysis.

To analyze the expression of HT genes, all available experimental microarray and proteomics data were analyzed [43–50]. Genes were considered to be expressed, if significant expression was observed in microarray experiments, or corresponding proteins were found in proteome analyses.

### Dependence on the database composition

To evaluate the annual change of the predicted HGT rate in the *Methanosarcina* spp., all archaeal completely sequenced genomes were extracted from Genbank, and the year of sequencing was recorded for each genome. A series of year-stamped databases based on BLASTP non-redundant database were made from year 2001 through 2011, in which all archaea that were sequenced after the given year were masked. BLASTP searches for each GOP member were made with default parameters against year-stamped databases.

## Availability of supporting data

The data sets supporting the results of this article are available online in the Dryad data repository under doi: http://dx.doi.org/10.5061/dryad.j69p2 [78], and other datasets supporting the results of this article are provided with Additional files.

## Additional files

Additional file 1:
**List of HT genes found in** ***Methanosarcina*** **spp.**
Additional file 2:
**ZIP archive with PL/SQL scripts used to assemble groups of orthologous proteins.**
